# Real-Time Motion Tracking for Mobile Augmented/Virtual Reality Using Adaptive Visual-Inertial Fusion

**DOI:** 10.3390/s17051037

**Published:** 2017-05-05

**Authors:** Wei Fang, Lianyu Zheng, Huanjun Deng, Hongbo Zhang

**Affiliations:** 1 School of Mechanical Engineering and Automation, Beihang University, Xueyuan Road, Haidian District, Beijing 100191, China; wfang@buaa.edu.cn (W.F.); zhanghongbo@buaa.edu.cn (H.Z.); 2 Beijing Baofengmojing Technologies Co., Ltd., Zhichun Road, Haidian District, Beijing 100191, China; deng_bu@aliyun.com

**Keywords:** real-time motion tracking, adaptive filter, visual-inertial fusion, mobile AR/VR, pose estimation

## Abstract

In mobile augmented/virtual reality (AR/VR), real-time 6-Degree of Freedom (DoF) motion tracking is essential for the registration between virtual scenes and the real world. However, due to the limited computational capacity of mobile terminals today, the latency between consecutive arriving poses would damage the user experience in mobile AR/VR. Thus, a visual-inertial based real-time motion tracking for mobile AR/VR is proposed in this paper. By means of high frequency and passive outputs from the inertial sensor, the real-time performance of arriving poses for mobile AR/VR is achieved. In addition, to alleviate the jitter phenomenon during the visual-inertial fusion, an adaptive filter framework is established to cope with different motion situations automatically, enabling the real-time 6-DoF motion tracking by balancing the jitter and latency. Besides, the robustness of the traditional visual-only based motion tracking is enhanced, giving rise to a better mobile AR/VR performance when motion blur is encountered. Finally, experiments are carried out to demonstrate the proposed method, and the results show that this work is capable of providing a smooth and robust 6-DoF motion tracking for mobile AR/VR in real-time.

## 1. Introduction

Mobile augmented reality (AR) and virtual reality (VR) are cutting technologies nowadays, which could change many aspects of our existing ways of life. The objective of mobile AR is to render the virtual object in a real world context with an accurate posture, thus the system needs to know where the user is and what the user is looking at by mobile computing [1,2,3]. Mobile VR, on the other hand, allows different interactions and communications between the user and virtual world. If we want to create a feeling of presence in a synthetic VR environment, tracking the user’s posture is also essential. The information about the user’s 6-DoF pose allows the system to show the virtual environment from the user’s perspective [4,5]. Thus, pose tracking by calculating the location and the orientation of the user in real-time, is one of the most important issues in mobile AR/VR [6,7]. However, due to the limited computing ability of mobile devices, the real-time motion tracking for mobile AR/VR is still a bottleneck.

Currently, motion tracking methods for mobile AR/VR can be classified into three categories: marker-based [8,9,10], model-based [11,12,13] and markerless-based [14,15]. The marker-based or model-based methods can only perform 6-DoF tracking with certain prior knowledge about the scene, while the markerless-based motion tracking can work within unprepared environments. Consequently, the markerless tracking method would be a more popular one for mobile AR/VR in future. However, due to heavy computing demands and unpredictable environments, the applicability and robustness of the real-time 6-DoF markerless motion tracking still need further research for mobile AR/VR. Especially in the VR scene, the jitter and latency between consecutive arriving poses would degrade the user experience severely.

Given the light weight and power consumption of mobile terminals, a markerless real-time motion tracking method for mobile AR/VR is proposed in this paper. To the best of our knowledge, this is the first paper to address the jitter and latency both for mobile AR and VR in visual-inertial fusion, especially for a higher frame-rate requirements in mobile VR. The main contributions of the paper are as follows:

1. By combining a monocular camera and an inertial sensor, sensor-fusion based 6-DoF motion tracking for mobile AR/VR in real-time is realized.
2. To alleviate the jitter during the visual-inertial fusion, an adaptive filter framework is proposed to balance the jitter and latency phenomenon, enabling a real-time and smooth 6-DoF motion tracking for mobile AR/VR.

Before a description of the proposed motion tracking approach, we would like to introduce related works in Section 2. In Section 3, the materials and method of the proposed adaptive visual-inertial based motion tracking are detailed. Then, experiments are carried out to demonstrate the proposed method in Section 4. Finally, the discussion and conclusions of the work are provided in Section 5 and Section 6, respectively.

## 2. Related Works

As a typical markerless tracking method, simultaneous localization and mapping (SLAM) can perceive the 6-DoF pose of a moving camera within an unprepared environment, enabling mobile AR/VR applications without fiducial markers. Given the monocular camera-based visual-tracking for our sensor fusion, the publication of PTAM [16] is groundbreaking. It divides the tracking and mapping in parallel for a real-time operation. Nevertheless, this method does not consider the loop-closure during SLAM. Thus, which is only suitable for the real-time AR tracking in a small scene. Based on the PTAM framework, Mur-Artal et al. proposed ORB-SLAM [17], which became one of the most successful monocular SLAM methods until now. By including the covisibility graph constraints, it can maintain a large global map during the tracking. Moreover, the loop-closure and re-localization modules make this method more powerful. Instead of the feature-based methods above, Silveira [18] proposed a direct SLAM method that addressed the matching problem according to the photometrics of an entire image. Then, DTAM [19] and LSD-SLAM [20] were proposed; these tracking methods are based on the minimization of the photometric pixel values instead of the feature-based matching, thus possessing larger computational requirements. The so-called direct method is able to generate a dense map by tracking a moving camera. To merge the mutual advantages of the feature-based and direct method, some hybrid SLAM methods [21,22] were proposed.

In addition, to implement the SLAM technology for mobile AR/VR, real-time performance for camera tracking is crucial. For mobile AR, the frequency of arriving poses should reach the normal video frame-rate (25 Hz), which is usually defined as a standard regulation for a real-time performance. This standard is also considered in this paper for real-time performances other than in mobile VR. Because the real-time performance in mobile VR is superior to conventional scenarios, the arriving frequency of the 6-DoF motion tracking should reach 60 Hz or more. Only in this way, the participant within the VR environment can enjoy a comfortable experience. Otherwise, the delay phenomenon would cause the user disgust in VR environments. However, the direct SLAM method involves the photometric error of the entire image, performing a dense (all pixel in the image) or a semi-dense (high gradient areas) reconstruction while tracking the camera, and the GPU acceleration is needed for a real-time performance due to the computational cost involved [23]. The computing ability of consumer mobile devices is insufficient for the real-time camera tracking by a direct method. In mobile AR/VR, we focus on the real-time camera tracking instead of the dense map. Besides it has high efficiency capacity, and the feature-based SLAM is also considered as more accurate than direct SLAM [17]. Therefore, the feature-based SLAM framework is chose as the visual-based tracking method in this paper.

To achieve real-time motion tracking by the feature-based method, Xu et al. [24] proposed a motion tracking method based on pre-captured reference images, which can prevent a gradual increase in the camera position error and address the wide baseline correspondence problem. Lee et al. [25] described a real-time motion tracking framework, focusing on the integration of nonlinear filters to achieve a robust tracking and a scalable feature mapping, which can be extended to a larger environment. However, the experiments of these real-time tracking methods were only performed on PC. In order to make a real-time motion tracking for mobile devices, Wei et al. [26] designed a fast and compact key frame search algorithm using the modified vector of locally aggregated descriptors, which can reduce the memory usage on mobile devices significantly. Chen et al. [27] proposed an improved mobile AR system based on the combination of ORB feature and optical flow. Then the RANSAC method was used to choose good features, and thus the homography matrix is obtained. Although these feature-based tracking methods can speed up the processing efficiency in mobile terminals, these methods often lead to some unsteadiness when they suffer from different motion situations. To alleviate the unstability phenomenon, Wang et al. [28] proposed a hybrid method for mobile AR by integrating feature points and lines, and these hybrid features are applied to fulfill the real-time motion tracking. Usually, the stable feature lines enable more stable and smoother camera trajectories.

However, these visual-only-based tracking approaches still suffer from poor feature or motion blur, making salient image features untractable. Moreover, to alleviate the dizziness phenomenon in a virtual environment, the frame-rate requirement for mobile VR is much higher than in mobile AR. Thus, the visual-only based tracking methods mentioned above are not appropriate for both mobile AR and VR. In order to improve the robustness and frame-rate of motion tracking for mobile AR/VR, other sensors should be applied to assist. As a primary motion capture sensor, the inertial sensor can provide high-frequency and passive measurements for pose estimation. Some researchers have summarized the 6-DoF motion tracking by visual-inertial fusion [29,30].

According to different fused frameworks, the sensor fusion solutions can be grouped into tightly coupled and loosely coupled. The tightly coupled approaches [31,32] can perform systematic fusion of the visual and Inertial Measurement Unit (IMU) measurements, usually leading to an additional complexity, while the loosely coupled approaches optimize the visual tracking and IMU tracking separately, thus presenting lower computational complexity. To perform the real-time motion tracking for mobile AR/VR, the efficiency of the loosely coupled based method is given greater attention in this paper.

The loosely coupled approaches consist of a standalone visual-based pose estimation module (such as PTAM [16], ORB-SLAM [17], LSD-SLAM [20]) and a separate IMU propagation module. Konolige et al. [33] integrated IMU measurements as independent inclinometer and relative yaw measurements into an optimization framework using stereo vision measurements. In contrast, Weiss et al. [34] used an individual visual-based pose estimation to correct the IMU propagation, but this method mainly paid attention to the scale estimation for unmanned aerial vehicles, and it did not consider the jitter and latency for possible mobile AR/VR applications. Tomazic et al. [35] proposed a fusion approach combining visual odometry and an inertial navigation on mobile devices, but this method is inclined to drift due to the lack of optimization at the back-end. Kim et al. [36] proposed an inertial and landmark-based integrated navigation method for poor vision environments. With the help of the inertial sensor, the system can provide reliable navigation when the number of landmarks is not sufficient for visual navigation. However, the dependency on the landmarks limits its adaptability. Li et al. [37] proposed a novel system for pose estimation using visual and inertial data, and only a three-axis accelerometer and colored marker are used for a 6-DoF motion tracking. Nevertheless, the pose calculation process is carried out on the server side for real-time performance, not within the mobile terminals. In summary, though these sensor fusion methods can perform a robust 6-DoF motion tracking, limited attention has been paid to real-time and smooth 6-DoF tracking, which may result in jitter and latency phenomenon for mobile AR/VR by fusing heterogeneous sensors, making these method not suitable for mobile AR/VR applications.

## 3. Materials and Methods

### 3.1. Platform and System Description

**Figure 1:**
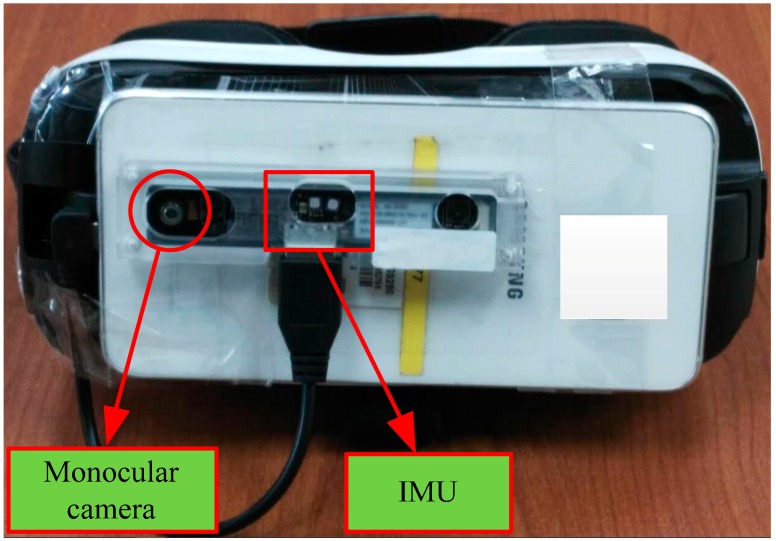
The experimental platform for real-time motion tracking.

The experimental platform used in this paper is illustrated in Figure 1. In order to improve the field of view (FOV) of the camera, an external sensor module containing a wide-angle monocular camera and an IMU is applied in this work. The monocular camera can obtain the image stream with 640 × 480 pixels at 30 fps, and the IMU can output the linear acceleration and angular velocity with 250 Hz. All the original data streams are ported to the mobile device (SAMSUNG S6, CPU: Exynos 7420 (1.5 GHz)) by USB 3.0 for post-processing, and the image and IMU measurement streams are associated with the timestamp. In order to evaluate the performance of the proposed method, no GPU or other acceleration methods to speed up the motion tracking are used in this paper.

**Figure 2:**
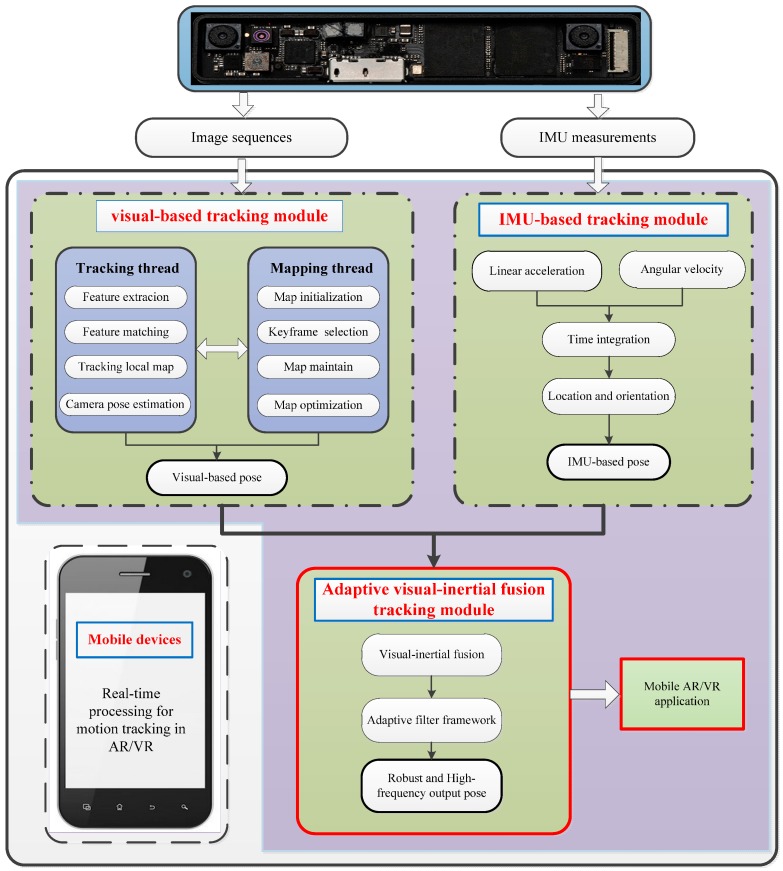
The system description of the adaptive visual-inertial fusion for mobile AR/VR.

Given the data streams from the experimental platform, the sequential images and IMU measurements arrive within a certain time interval. They are associated with the timestamp, and then an adaptive visual-inertial based motion tracking for mobile AR/VR can be performed. The detailed description of the proposed method is depicted in Figure 2. It includes three main modules: a visual-based tracking module, an IMU-based tracking module and an adaptive visual-inertial fusion tracking module. The visual-based tracking module can provide the pose estimation with high accuracy but low frequency, while the IMU-based tracking module generates high frequency pose estimations. Then, the adaptive visual-inertial fusion module is used to combine mutual advantages. Finally, a real-time 6-DoF motion tracking is obtained for mobile AR/VR without the jitter and latency phenomenon.

### 3.2. Monocular Visual and IMU Based Tracking

#### 3.2.1. Monocular Parameter Calibration

Given a homogeneous point in the world frame $P_{w}={\left[x_{w},y_{w},z_{w},1\right]}^{T}$, the corresponding undistorted homogeneous image point is $m={\left[u,v,1\right]}^{T}$. Thus, the relationship between a 3D point $P_{w}$ and its image projection m is given by:

(1) $$sm=K\left[R\;t\right]P_{w}=\left[\begin{array}{ccc} f_{x} & 0 & u_{0}\\ 0 & f_{y} & v_{0}\\ 0 & 0 & 1 \end{array}\right]\left[R\;t\right]P_{w}$$

where s represents the non-zero scale factor, K denotes the intrinsic parameter matrix of the camera, $\left(u_{0},v_{0}\right)$ is the coordinate of the principal point, and $f_{x}$ and $f_{y}$ are the scale factors in image u and v axes. [R     t] is the transformation from the world frame to the camera frame.

As a wide-angle monocular camera in the sensor module, the radial distortion plays a dominant role. Thus, only the radial distortion coefficient $\left(k_{1},k_{2}\right)$ of the camera lens is be taken into account. Let (u,v) be the ideal image coordinates (distortion free), and $\left(u_{d},\;v_{d}\right)$ is the corresponding real observed image coordinates (distorted). The center of the radial distortion is assumed to be the principal point. Then:

(2) $$\{\begin{array}{c} u_{d}=u+(u-u_{0})(k_{1}r^{2}+k_{2}r^{4})\\ v_{d}=v+(v-v_{0})(k_{1}r^{2}+k_{2}r^{4}) \end{array}$$

where $r=\sqrt{{\left(u_{d}-u_{0}\right)}^{2}+{\left(v_{d}-v_{0}\right)}^{2}}$ is the distorted radius, and the intrinsic parameter of the monocular camera is obtained by the Zhang method [38].

#### 3.2.2. Visual-Based Tracking

According to the calibrated parameters of the wide-angle monocular camera in Section 3.2.1, the visual-based tracking can be carried out with successive arriving images. The open-source ORB-SLAM [17] provides a valuable visual-SLAM framework for our work. Moreover, the feature-extraction constraint and keyframe strategy are reinforced in this work for a more efficient and robust performance.

The tracking system maintains a complementary global map of 2D-3D correspondences through aggregated keyframe data, which is also useful for optimization. Thus, the inserted strategy of keyframe plays an important role for the tracking stability and consistency. On the basis of the keyframe selection in ORB-SLAM, an area-based strategy is appended according to the feature point distribution on the keyframe. Supposed that a series of feature points $(u_{i},v_{i}),i=1,\cdots ,n$ on the $j^{th}$ keyframe, the external bounding polygon POL is determined. Thus, the feature distribution condition on the image threshold of the areaRatio is defined as:

(3) $$areaRatio=\frac{POL_{Area}}{I_{Width}\cdot I_{Height}}$$

where the $POL_{Area}$ is the area of polygon POL, while the total image area is computed by $I_{Width}\cdot I_{Height}$. In our tracking system, the threshold is set to 0.5, and a new keyframe is inserted when the areaRatio of current image is lower than 0.5.

**Figure 3:**
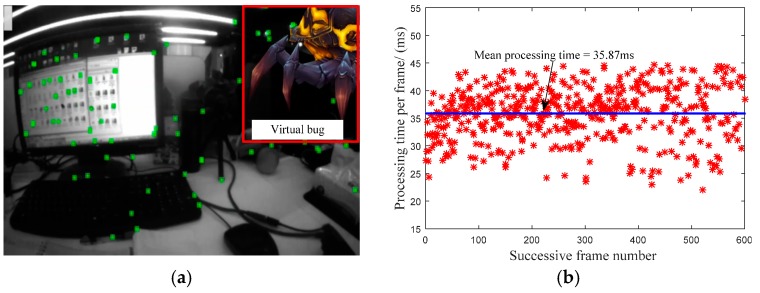
Efficiency statistics for visual-based tracking: (**a**) typical visual-based tracking scene; (**b**) processing time per frame.

To verify the processing efficiency of our visual-based tracking in mobile devices, the total processing time from the feature-extraction in front-end to the pose optimization in back-end for every incoming frame is collected. The tracking scene is shown Figure 3a, where the virtual bug in the image is used to demonstrate the successful motion tracking in mobile devices. The processing time per-frame for successive 600 frames is depicted in Figure 3b. We can find that the mean processing time for every frame is about 35.87 ms, and the maximum and minimum processing time is 44.74 ms and 22.07 ms, respectively. The difference is mainly depended on the number of the extracted features from the input image. Generally speaking, the mean motion tracking efficiency can reach nearly real-time performance within the mobile device (about 28 Hz).

#### 3.2.3. Process Model for Visual-Inertial Fusion

**Figure 4:**
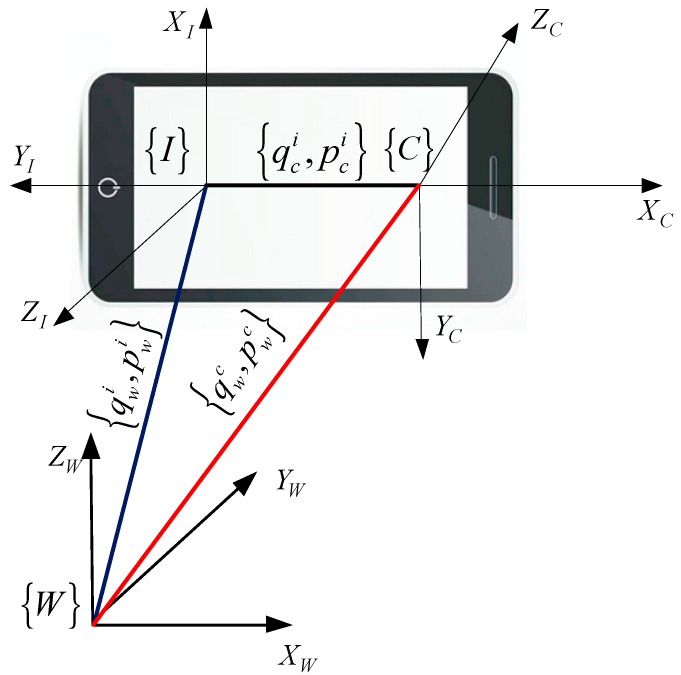
Visual-inertial coordinate frames within mobile devices.

The main purpose of this work is to estimate the 6-DoF of mobile devices in unprepared environments. Given the mobile experimental platform, the relationships of different frames are shown in Figure 4, where the IMU frame {I} and the camera frame {C} are rigidly connected, and the world frame is denoted as {W}. The quaternion and position pair $\left\{q_{w}^{i},\;p_{w}^{i}\right\}$ denotes the transformation of the IMU in the global frame, while $\left\{q_{w}^{c},\;p_{w}^{c}\right\}$ represents the transformation of the camera with respect to the global frame. The pair $\left\{q_{i}^{c},\;p_{i}^{c}\right\}$ denotes the orientation and position of the IMU in the camera frame, this is a fixed value when the sensor module developed and which can be calibrated by the method [39] in advance.

The measurements of the IMU contain a certain bias b and a white Gaussian noise n. Thus, the real angular velocity ω and the real acceleration a related with gyroscope and accelerometer measurements are obtained, respectively:

(4) $$\omega_{m}=\omega +b_{\omega }+n_{\omega }\;a_{m}=a+b_{a}+n_{a}$$

The subscript m denotes the measured value, and dynamics of the non-static bias b is modeled as a random process.

The IMU state vector comprises of its position, velocity ($v_{w}^{i}$), orientation in the world frame and the biases of the gyroscope and accelerometer. To make the posture fusion from the visual and inertial sensor, the transformation from the IMU frame to the camera frame is also included in the state vector. Thus, the state vector X is obtained:

(5) $$X=\left\{{p_{w}^{i}}^{T}\;{v_{w}^{i}}^{T}\;{q_{w}^{i}}^{T}\;{b_{\omega }}^{T}\;b_{a}^{T}\;{p_{i}^{c}}^{T}\;{q_{i}^{c}}^{T}\right\}$$

Then, the data-driven dynamic model is represented by the following differential equations:

(6) $$\begin{array}{l} {\dot{p}}_{w}^{i}=v_{w}^{i}\;,\;{\dot{v}}_{w}^{i}=C_{\left(q_{w}^{i}\right)}^{T}a-g,\;{\dot{q}}_{w}^{i}=\frac{1}{2}\Omega \left(\omega \right)q_{w}^{i}\\ \;{\dot{b}}_{\omega }=n_{b\omega }\;,\;{\dot{b}}_{a}=n_{ba},\;{\dot{p}}_{i}^{c}=0,\;{\dot{q}}_{i}^{c}=0 \end{array}$$

where, $C_{\left(q_{w}^{i}\right)}^{T}$ is the rotational matrix corresponding to the quaternion $q_{w}^{i}$, and g is the gravity vector in the world frame {W}. Ω(ω) is the quaternion multiplication matrix of ω, and $\Omega \left(\omega \right)=\left[\begin{array}{cc} -{\left\lfloor \omega \right\rfloor }_{\times } & \omega^{T}\\ -\omega^{T} & 0 \end{array}\right]$, ${\left\lfloor \omega \right\rfloor }_{\times }=\left[\begin{array}{ccc} 0 & -\omega_{3} & \omega_{2}\\ \omega_{3} & 0 & -\omega_{1}\\ -\omega_{2} & \omega_{1} & 0 \end{array}\right]$ is the skew-symmetric matrix. Assuming $\bar{q}={\left(q_{0},q\right)}^{T}$ is a unit quaternion and its corresponding rotational matrix is represented as $C_{\bar{q}}$. These two orientation representations can be related as below:

(7) $$C_{\bar{q}}=(2{q_{0}}^{2}-1)I_{3}-2q_{0}{\left\lfloor q\right\rfloor }_{\times }+2qq^{T}$$

Since the mean value of the noise is assumed to be zero, the IMU nominal-state kinematics is obtained:

(8) $$\begin{array}{l} {\hat{\dot{p}}}_{w}^{i}={\hat{v}}_{w}^{i},\;{\hat{\dot{v}}}_{w}^{i}=C_{\left({\hat{q}}_{w}^{i}\right)}^{T}\left(a_{m}-{\hat{b}}_{a}\right)-g\\ \;{\hat{\dot{q}}}_{w}^{i}=\frac{1}{2}\Omega \left(\omega_{m}-{\hat{b}}_{\omega }\right){\hat{q}}_{w}^{i}\\ {\hat{\dot{b}}}_{\omega }=0\;,\;{\hat{\dot{b}}}_{a}=0,\;{\hat{\dot{p}}}_{i}^{c}=0,\;{\hat{\dot{q}}}_{i}^{c}=0 \end{array}$$

and, the error quaternions are defined as follows:

(9) $$\begin{array}{l} \delta q_{w}^{i}=q_{w}^{i}⊗{\hat{q}}_{w}^{i-1}\approx {\left[\frac{1}{2}\delta {\theta_{w}^{i}}^{T}\;1\right]}^{T}\\ \delta q_{i}^{c}=q_{i}^{c}⊗{\hat{q}}_{i}^{c-1}\approx {\left[\frac{1}{2}\delta {\theta_{i}^{c}}^{T}\;1\right]}^{T} \end{array}$$

In order to minimize the dimension of the filter state vector, the 21-elements of error state vector are determined as:

(10) $$\tilde{x}=\left\{\Delta {p_{w}^{i}}^{T}\;\Delta {v_{w}^{i}}^{T}\;\delta {\theta_{w}^{i}}^{T}\;\Delta {b_{\omega }}^{T}\;\Delta {b_{a}}^{T}\;\Delta {p_{i}^{c}}^{T}\;\delta {\theta_{i}^{c}}^{T}\right\}$$

Given the error state filter formulation, the relationship between the true state x, nominal state $\hat{x}$, and error state $\tilde{x}$ is:

(11) $$x=\hat{x}+\tilde{x}$$

Then, with $\hat{\omega }=\omega_{m}-{\hat{b}}_{\omega }$ and $\hat{a}=a_{m}-{\hat{b}}_{a}$, the differential equations for the continuous time error state are obtained:

(12) $$\begin{array}{l} \;\Delta {\dot{p}}_{w}^{i}=\Delta v_{w}^{i}\\ \;\Delta {\dot{v}}_{w}^{i}=-C_{\left({\hat{q}}_{w}^{i}\right)}^{T}{\left\lfloor \hat{a}\right\rfloor }_{\times }\delta \theta -C_{\left({\hat{q}}_{w}^{i}\right)}^{T}\Delta b_{a}-C_{\left({\hat{q}}_{w}^{i}\right)}^{T}n_{a}\\ \;\delta {\dot{\theta }}_{w}^{i}=-{\left\lfloor \hat{\omega }\right\rfloor }_{\times }\delta \theta -\Delta b_{\omega }-n_{\omega }\\ \Delta {\dot{b}}_{\omega }=n_{b_{\omega }},\;\Delta {\dot{b}}_{a}=n_{b_{a}},\;\Delta {\dot{p}}_{i}^{c}=0\;,\;\Delta {\dot{\theta }}_{i}^{c}=0 \end{array}$$

Within the filter prediction stage, the inertial measurements for state propagation are obtained in discrete form. Thus the signals from gyroscope and accelerometer are assumed to sample with a certain time interval, and the nominal state is obtained with the numerical integration of the 4th Runge-Kutta method. By stacking the differential equations for error state, the linearized continuous time error state equation is given:

(13) $$\dot{\tilde{x}}=F_{c}\tilde{x}+G_{c}n$$

where the noise vector $n={\left[n_{a}^{T},n_{b_{a}}^{T},n_{\omega }^{T},n_{b_{\omega }}^{T}\right]}^{T}$, and $F_{d}$ is acquired by digitizing $F_{c}$ by the following Taylor series:

(14) $$F_{d}=\exp(F_{c}\Delta t)=I_{d}+F_{c}\Delta t+\frac{1}{2}F_{c}^{2}\Delta t^{2}+\cdots$$

Analysis of the $F_{d}$ exponents reveal a repetitive and sparse structure [40]. With $Q_{c}$ being the noise covariance matrix $Q_{c}=diag(\sigma_{n_{a}}^{2}\cdot I,\sigma_{n_{b_{a}}}^{2}\cdot I,\sigma_{n_{\omega }}^{2}\cdot I,\sigma_{n_{b_{\omega }}}^{2}\cdot I)$, the covariance matrix $Q_{d}$ is obtained by the discretization of $Q_{c}$:

(15) $$Q_{d}=\int_{\Delta t}F_{d}\left(\tau \right)G_{c}Q_{c}G_{C}^{T}F_{d}{\left(\tau \right)}^{T}d\tau$$

Thus, the covariance matrix is computed:

(16) $$P_{k|k-1}=F_{d}P_{k-1|k-1}{F_{d}}^{T}+Q_{d}$$

Therefore, with the discretized error state propagation and error noise covariance matrices, the state can be propagated as follows:

(a) When IMU data $\omega_{m}$ and $a_{m}$ arrived in a certain sample frequency, the state vector is propagated using numerical integration on Equation (8).
(b) Calculate $F_{d}$ and $Q_{d}$.
(c) Compute the propagated state covariance matrix according to the Equation (16).

### 3.3. Adaptive Visual-Inertial Fusion for Mobile AR/VR

#### 3.3.1. Measurement Model for Visual-Inertial Fusion

According to the visual-based tracking method discussed in Section 3.2.2, the location and orientation of the camera are obtained. As an inertial sensor, the integrated drift over time may lead the motion tracking collapsed due to the bias and noise inherent. Therefore, postures of the camera from visual-based tracking are applied as measurements in the Extended Kalman Filter framework. For the camera position measurement $p_{w}^{c}$, the following measurement model is obtained:

(17) $$z_{p}=p_{w}^{c}=p_{w}^{i}+C_{\left(q_{w}^{i}\right)}^{T}p_{i}^{c}+n_{p}$$

where $C_{\left(q_{w}^{i}\right)}$ and $n_{p}$ is the IMU’s attitude in the world frame and the measurement position noise, respectively. And the position error is defined as:

(18) $${\tilde{z}}_{p}=z_{p}-{\hat{z}}_{p}$$

Equation (18) can be linearized as follows:

(19) $${\tilde{z}}_{pl}=H_{p}\tilde{x}$$

At the same time, the orientation of camera is derived by the error quaternion. The rotation from camera frame to world frame yielded from visual-based tracking is $q_{w}^{c}$, thus:

(20) $$z_{q}=q_{w}^{c}=q_{i}^{c}⊗q_{w}^{i}$$

Therefore, the error measurement of orientation is acquired:

(21) $${\tilde{z}}_{q}=z_{q}-{\hat{z}}_{q}=z_{q}⊗{{\hat{z}}_{q}}^{-1}=(q_{i}^{c}⊗q_{w}^{i})⊗(q_{i}^{c}⊗{\hat{q}}_{w}^{i})$$

Finally, the measurements are stacked next:

(22) $$\left[\begin{array}{c} {\tilde{z}}_{p}\\ {\tilde{z}}_{q} \end{array}\right]=\left[\begin{array}{c} H_{p}\\ H_{q} \end{array}\right]\tilde{x}$$

where $H_{p}$ and $H_{q}$ are the Jacobian matrix corresponding to the location and orientation, respectively.

According to the above process, the measurement update can be realized. And the total fusion process is summarized as Algorithm 1.

**Algorithm 1.** Visual-inertial motion tracking process.01. Initialize ${\hat{x}}_{0|0}$, ${\tilde{x}}_{0|0}$ and $P_{0|0}$02. **for**
k=1,⋯ do03. { **Time update**:04.   Compute $F_{d}$ and $Q_{d}$, ${\tilde{x}}_{k|k-1}=0_{21\times 1}$, $P_{k|k-1}=F_{d}P_{k-1|k-1}{F_{d}}^{T}+Q_{d}$05.   Compute ${\hat{x}}_{k|k-1}$ with the 4th Runge Kutta integration06.     **if** Pose from visual-based arrived07.      {**Measurement update**:08.       Compute the residual: $\tilde{z}=z-\hat{z}$, Kalman gain: $K_{k}=P_{k|k-1}H^{T}{\left(HP_{k|k-1}H^{T}+R\right)}^{-1}$;09.       Compute the correction: ${\tilde{x}}_{k|k}={\tilde{x}}_{k|k-1}+K_{k}\tilde{z}$, $P_{k|k}=(I_{d}-K_{k}H)P_{k|k-1}{\left(I_{d}-K_{k}H\right)}^{T}+K_{k}R{K_{k}}^{T}$;10.       Use ${\tilde{x}}_{k|k}$ to correct state estimate and the obtain ${\hat{x}}_{k|k}$}11.     **end**12. **end** }

With the aforementioned visual-inertial fusion, the frequency of 6-DoF pose estimation can be improved to satisfy the real-time performance for mobile AR/VR. However, due to the fact output poses derived from two heterogeneous sensors have different precisions and frequencies, this results in jitter phenomena during sensor fusion. In order to alleviate the jitter phenomenon for mobile AR/VR, an adaptive filter framework is proposed to smooth the jitter phenomenon without latency in following sections.

#### 3.3.2. Quaternion-Based Linear Filter Framework

In 6-DoF posture, the quaternion is used to represent 3D rotation, and the unit quaternion $\bar{q}={\left(q_{0},q\right)}^{T}$ can be transformed into the following form:

(23) $$\bar{q}=\cos\frac{\theta }{2}+\left(\sin\frac{\theta }{2}\right)\vec{u}$$

where $\cos\frac{\theta }{2}=q_{0}$, $\sin\frac{\theta }{2}=\sqrt{q\cdot q}$, and $\vec{u}=\frac{q}{\sqrt{q\cdot q}}$ when q⋅q is not equal zero [41]. This expression describes the relationship between the quaternion and a rotation in 3D space. In this case, θ represents the magnitude of rotation around an axis $\vec{u}$. Thus, for any unit quaternion $\bar{q}={\left[w\;x\;y\;z\right]}^{T}$, the corresponding rotation θ and axis $\vec{u}$ are obtained by Equation (24):

(24) $$\{\begin{array}{c} \theta =2\cdot a\cos\left(w\right)\\ \vec{u}=\left(\sin\frac{\theta }{2}\right)\cdot \left[x\;y\;z\right] \end{array}$$

Given the high-frequency posture from the visual-inertial fusion, the change between continuous arriving orientations is considered to be small enough. Thus, a linear quaternion interpolation filter is applied in the paper. With the help of Equation (24), the quaternion $q_{i}\;\left(i=0,\cdots ,n\right)$ is converted to be a set $\left(\theta_{i},{\vec{u}}_{i}\right)$. For a certain filter coefficient $\beta_{i}\in \left[0,1\right]$, the current $i^{th}$ filtered posture $\left\{q_{i}^{filter},p_{i}^{filter}\right\}$ is obtained:

(25) $$\{\begin{array}{c} q_{i}^{filter}:\left(\theta_{i},{\vec{u}}_{i}\right)=\beta_{i}\left(\theta_{i},{\vec{u}}_{i}\right)+\left(1-\beta_{i}\right)\left(\theta_{i-1},{\vec{u}}_{i-1}\right)\\ p_{i}^{filter}:p_{i}=\beta_{i}p_{i}+\left(1-\beta_{i}\right)p_{i-1} \end{array}$$

where $\left\{q_{0},p_{0}\right\}$ is defined as the initial arriving pose, and the adaptive coefficient $\beta_{i}$ imposes a linear filter effect to continuous arriving poses. If the value $\beta_{i}$ is set close to 0, the current filtered pose $\left\{q_{i}^{filter},p_{i}^{filter}\right\}$ is derived from previous ones, and the jitter phenomenon can be seriously smoothed under this circumstance. However, due to the fact the current filtered pose for mobile AR/VR relies heavily on the previous ones, the latency phenomenon is obvious. If the value $\beta_{i}$ is set close to 1, the arriving pose from visual-inertial fusion is fed to the mobile AR/VR almost directly. Thus, the current filtered pose $\left\{q_{i}^{filter},p_{i}^{filter}\right\}$ owns no latency but suffering from obvious jitter derived from the heterogeneous sensor fusion. Therefore, how to perform a real-time motion tracking by balancing the jitter and latency under different situations automatically is a key issue for mobile AR/VR.

#### 3.3.3. Different Motion Situations Analysis

Given the existed monocular camera and inertial sensor, the frame-rate of arriving images and IMU measurements is constant, leading to a constant time interval between adjacent arriving poses from the visual-inertial fusion. Thus, the location changes of adjacent postures are applied to distinguish different motion situations during the real-time 6-DoF motion tracking. For the $i^{th}$ arriving pose, the real-time Euclidean distance $d_{i}$ of the current posture is defined:

(26) $$d_{i}={\left\|p_{i-1}^{filter}-p_{i}\right\|}_{2}$$

where $p_{i-1}^{filter}$ is the previous ${\left(i-1\right)}^{th}$ filtered location from Equation (25), and $p_{i}$ is the $i^{th}$ arriving location. As the tracking goes on, a serial of Euclidean distances $d_{i}$ are obtained, thus a real-time updated distance range set $\left(D_{iMax},D_{iMin}\right)$ is acquired:

(27) $$\left\{\left(D_{iMax}，D_{iMin}\right)：D_{iMax}=\max\left(d_{1},d_{2,}\cdots ,d_{i}\right),D_{iMin}=\min\left(d_{1},d_{2,}\cdots ,d_{i}\right)\right\}$$

Then, in order to evaluate different motion situations generally, a normalized distance $p_{ith}\;$ is defined:

(28) $$p_{ith}=\frac{d_{i}-D_{iMin}}{D_{iMax}-D_{iMin}}\;p_{ith}\in \left[0,1\right]$$

**Figure 5:**
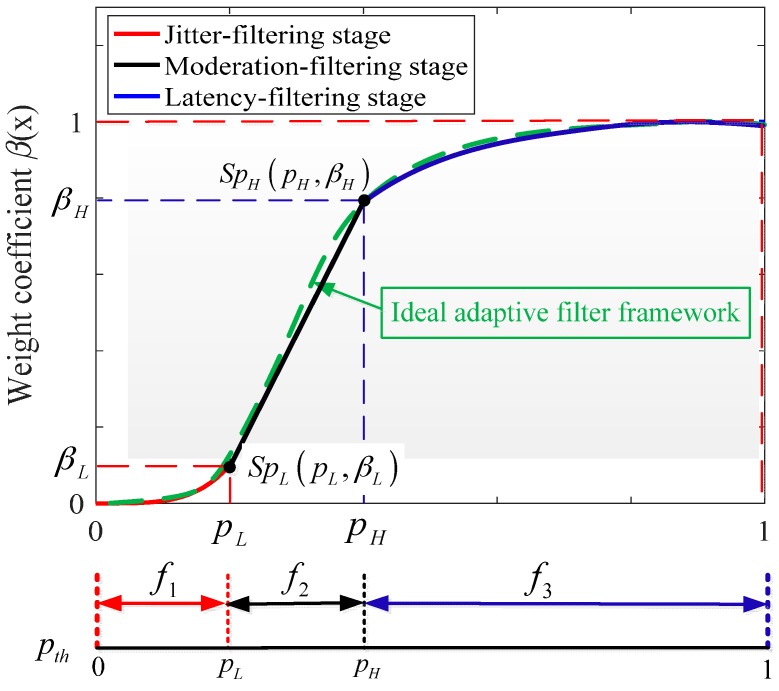
The schematic diagram of adaptive filter framework.

If the normalized distance $p_{ith}$ is close to 0, illustrating that the mobile device is in an almost static state. Otherwise, the mobile device is in a relative fast motion situation when $p_{ith}$ is close to 1. Thus, according to different normalized distances $p_{ith}$, an adaptive filter framework shown in Figure 5 is built to adjust the filtering weight coefficient $\beta_{i}$ automatically, balancing the jitter and latency for mobile AR/VR under different motion situations.

Ideally, the adaptive filter framework should be continuous enough under different motion situations, as the green dotted line shown in Figure 5. However, it is not possible to obtain an ideal and optimal adaptive filter framework uniformly to address the jitter and latency phenomenon. For a simplifying assumption, two segmented points $Sp_{L}\left(p_{L},\beta_{L}\right)$ and $Sp_{H}\left(p_{H},\beta_{H}\right)$ are set to divide different motion situations in this paper, and then three corresponding segmented functions can be built to approximate the ideal adaptive filter framework, which are jitter-filtering, moderation-filtering and latency-filtering. As shown in Figure 5, given the jitter-filtering stage for example, the normalized distance $p_{ith}$ locates close to 0. Thus a certain range of $p_{ith}\in \left[0,p_{L}\right]$ is defined and the corresponding filter weight $\beta_{i}\in \left[0,\beta_{L}\right]$ is applied to perform the jitter-removing. Another segmented point to divide the moderation-filtering and latency filtering is $Sp_{H}\left(p_{H},\beta_{H}\right)$, distinguishing the moderate and fast motion situations.

The segmented functions $f_{1}$, $f_{2}$ and $f_{3}$ in Figure 5, corresponding to the jitter-filtering, moderation-filtering and latency-filtering stage, can be used to balance the jitter and latency for visual-inertial fusion in this paper. In order to illustrate the proposed adaptive filter framework further, detailed analyses of the proposed segmented strategies are as follows:

(a) *Jitter-filtering*: When the mobile AR/VR system is almost kept static or moves slowly, the change between adjacent poses can be almost ignored. Thus, the jitter phenomenon plays a dominant role at this scenario, while the latency phenomenon for mobile AR/VR can be neglected for users’ perception, and this stage is defined as jitter-filtering in this paper. At the same time, the real-time distances between successive arriving poses are small enough at this stage, meaning that the normalized distance $p_{ith}$ is close to 0.
(b) *Moderation-filtering*: When the motion situation of the mobile AR/VR system is under moderate motion situations, this stage is defined as moderation-filtering in our work with a moderate distance $p_{ith}$.
(c) *Latency-filtering*: When the mobile AR/VR system is encountered rapid motion, the change between adjacent arriving poses is drastic. The user would perceive the latency obvious when the pose cannot arrive timely. Thus, latency phenomenon plays a dominant role at this scenario for mobile AR/VR, while the jitter phenomenon can be neglected within a fast motion situation. This stage is defined as latency-filtering. Moreover, the real-time distances between successive arriving poses are relative large, meaning that the normalized distance $p_{ith}$ is close to 1.

#### 3.3.4. Adaptive Filter Framework Definition

**Figure 6:**
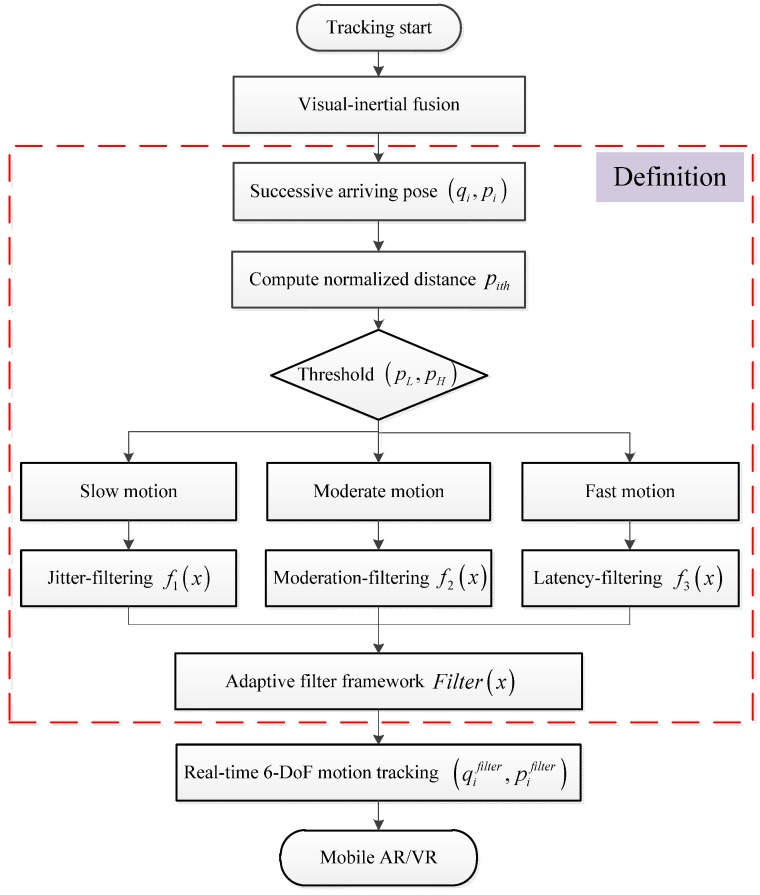
Flow diagram of the proposed adaptive filter framework.

According to the descriptions above, the normalized distance between adjacent postures can be applied to feed the proposed adaptive filter framework. Then, the corresponding filter stage is determined to address different motion situations. The flow diagram of the proposed adaptive filter framework is depicted in Figure 6.

To address the jitter and latency phenomenon effectively, quadratic functions are used in the jitter and latency stages to address extreme motion conditions smoothly. Moreover, in order to simplify the computing complexity on mobile terminals, a linear function is defined as the moderation-filtering to bridge the jitter-filtering and latency-filtering stages. The aim of these segmented framework is to approximate an ideal continuous framework, achieving a straightforward and suboptimal solution to balance the jitter and latency for mobile AR/VR.

Thus, given the jitter-filtering stage for example, the biquadratic function $f_{1}\left(x\right)$ can be derived from the origin (0,0) and segmented point $Sp_{L}\left(p_{L},\beta_{L}\right)$:

(29) $$f_{1}\left(x\right)=\frac{\beta_{L}}{{p_{L}}^{4}}x^{4},\;0\leq x=p_{ith}<p_{L}$$

Then, with another segmented point $Sp_{H}\left(p_{H},\beta_{H}\right)$ and end-point (1,1), the corresponding linear function $f_{2}\left(x\right)$ and quadratic fusion $f_{3}\left(x\right)$ are defined to address the moderation-filtering and latency-filtering stages as follows:

(30) $$\{\begin{array}{ll} f_{2}\left(x\right)=\frac{\beta_{H}-\beta_{L}}{p_{H}-p_{L}}x+\beta_{L}p_{H}-\beta_{H}p_{L}, & p_{L}\leq x=p_{th}<p_{H}\\ f_{3}\left(x\right)=-\frac{\beta_{H}-1}{{\left(p_{H}-1\right)}^{2}}{\left(x-1\right)}^{2}+1, & p_{H}\leq x=p_{th}\leq 1.0 \end{array}$$

Based on the segmented filter functions, Equations (29) and (30), a signum function (Equation (31)) is used to define a unified filter framework for different motion situations of the mobile device

(31) $$\mathrm{sgn}\left(x\right)=\{\begin{array}{ll} -1, & x<0\\ 0, & x=0\\ 1, & x>0 \end{array}$$

Then, combining Equations (29)–(31), the unified adaptive filter framework Filter(x) is obtained:

(32) $$Filter\left(x\right)=\frac{1-\mathrm{sgn}\left(x-p_{H}\right)}{2}\left\{\frac{1-\mathrm{sgn}\left(x-p_{L}\right)}{2}f_{1}\left(x\right)+\frac{1+\mathrm{sgn}\left(x-p_{L}\right)}{2}f_{2}\left(x\right)\right\}\;+\frac{1+\mathrm{sgn}\left(x-p_{H}\right)}{2}f_{3}\left(x\right)$$

with variable $x=p_{ith}$, substituting Equations (29)–(31) into Equation (32), and the adaptive filtering framework is established, where $p_{L}$ and $p_{H}$ are used to distinguish different motion situations. The experimental results (in the next Section 4.1) show that a suboptimal motion tracking performance can be achieved when $p_{L}$ and $p_{H}$ are set to 0.2 and 0.4 in the proposed tracking system, respectively. $\beta_{L}$ and $\beta_{H}$ are the corresponding weight coefficient to filter the arriving pose, and they are set to 0.1 and 0.9 to balance the jitter and latency for a good mobile AR/VR performance in the paper. Thus, according to the actual motion situations for the mobile AR/VR system unpredictability, the adaptive filter framework can balance the jitter and latency for a real-time motion tracking.

## 4. Experiments and Results

### 4.1. Adaptive Visual-Inertial Fusion Performance

**Figure 7:**
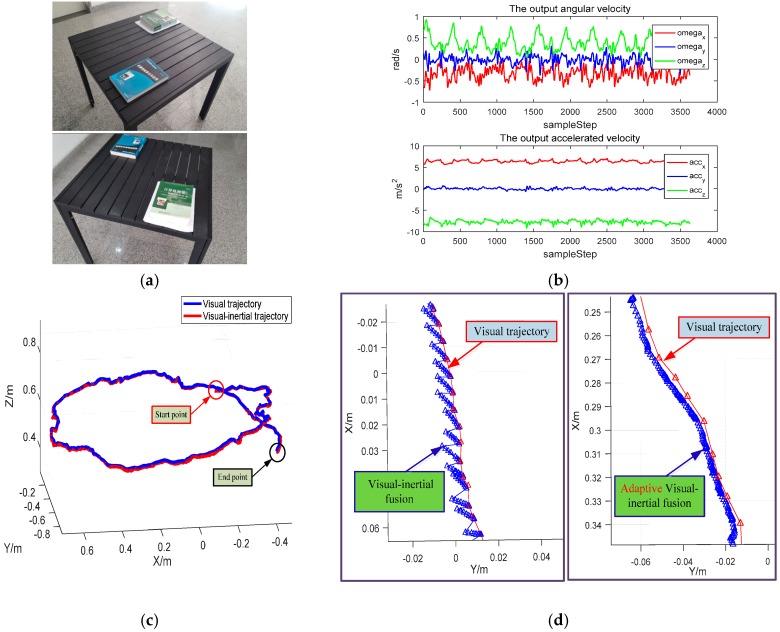
6-DoF motion tracking experiment by adaptive visual-inertial fusion: (**a**) typical images from the experimental scenes; (**b**) IMU outputs during the tracking process; (**c**) trajectories from different tracking methods; (**d**) comparisons from different visual-inertial fusion methods.

In order to evaluate the performance of the proposed real-time motion tracking for mobile AR/VR, a qualitative experiment is carried out. The mobile system is mounted on an operator and moved around the table. The typical images and the IMU measurements during the tracking process are depicted in Figure 7a,b, respectively. The recovered trajectories from visual-based and visual-inertial fusion based are illustrated in Figure 7c, and we can find that these two trajectories are close to each other, demonstrating the effectiveness of our proposed visual-inertial fusion method.

An enlarged image of the visual-inertial trajectory is depicted in Figure 7d, where the left side image illustrates the original visual-inertial fusion performance. The red line and triangles represent the visual-based trajectory and poses, while the blue line and triangles depicted the visual-inertial-based trajectory and poses. It is easy to find that the visual-inertial fusion method can provide a high-frequency arriving pose with the help of IMU, but it is inclined to drift due to the integrated error of IMU between the successive two visual frames. Thus, this may result in jitter phenomena in mobile AR/VR. With the proposed adaptive visual-inertial method, as shown at the right side in Figure 7d, a smooth trajectory with high-frequency pose outputs is realized by adaptive visual-inertial fusion. What is more, according to the credible pose estimation by IMU within a short time interval, the tracking stability can be improved when suffering from motion blur or weak texture.

**Figure 8:**
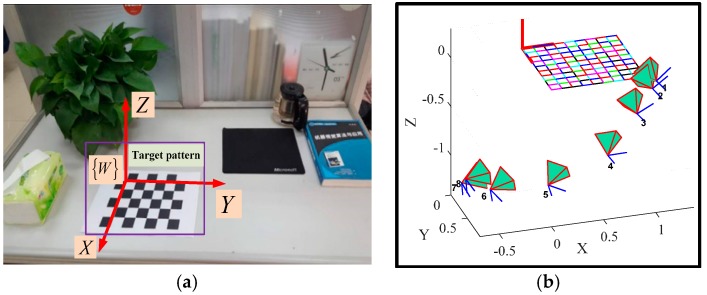
Quantitative evaluation of the proposed motion tracking method: (**a**) typical images from the comparing scene; (**b**) ground truth recovered from the standard target pattern.

In addition, another quantitative experiment is carried out to evaluate the proposed method further. A common target chessboard pattern is placed in a natural desktop scene, as shown in Figure 8a. The target pattern is a typical chessboard comprising of 6 × 7 squares (30 mm × 30 mm). Given the calibrated intrinsic parameters of the monocular camera in Section 3.2.1, if this target pattern is visible by the moving monocular camera, the 6-DoF motion trajectory of the camera can be derived from the standard target pattern (as shown in Figure 8b). The trajectory derived from the standard target pattern is considered to have a high accuracy, which can be applied as the ground truth for the proposed method.

**Figure 9:**
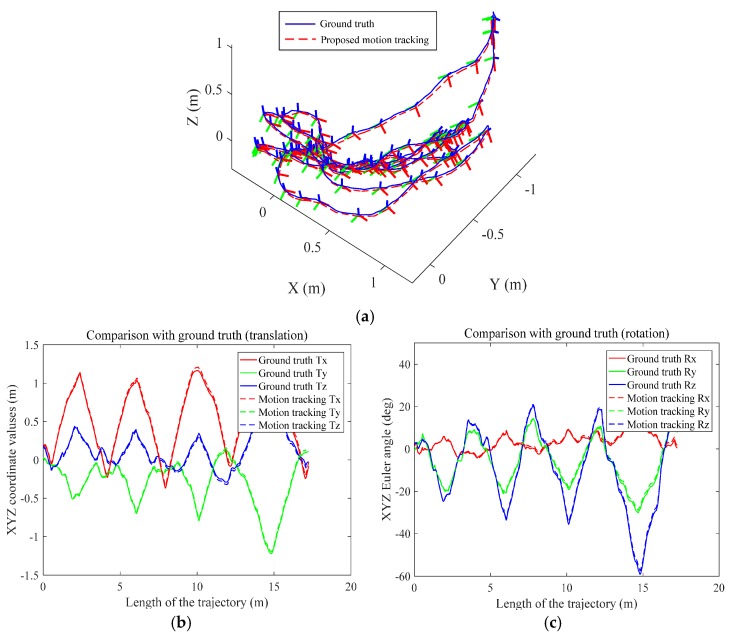
Accuracy evaluation of the proposed motion tracking method: (**a**) trajectory comparisons between ground truth and proposed motion tracking; (**b**) evaluation of the translational performance; (**c**) evaluation of rotational performance.

Then, the multi-sensor system is carried out back and forth around the desktop with the target pattern in the view, the total length of the ground truth trajectory derived from the target pattern is 17.28 m (as shown in Figure 9a). Then, aligning the ground truth and the proposed motion tracking trajectory by the target pattern and timestamp, the comparative trajectories are transformed and depicted in a common coordinate frame. The trajectory of the ground truth is illustrated in blue line within some sampling 6-DoF poses, and the trajectory of the proposed motion tracking is depicted in red dash line. The translational and rotational performances during the motion tracking experiment in different directions are shown in Figure 9b and Figure 9c, respectively. As can be seen, the plots are well superimposed as expected, which also demonstrates the accuracy of the proposed real-time motion tracking.

Given the error analyses of the contrast experiments above, the translational and rotational errors are calculated to evaluate the proposed real-time motion tracking performance. The average error is about 4 cm in translation, while the mean rotational error is about 0.7°. The maximum tracking error in Euclid is 14.72 cm (0.85% to the total length). More detailed illustrations of the error between two computed positions (translation and rotation) can be found in Table 1.

### 4.2. Real-Time Motion Tracking for Mobile AR

**Figure 10:**
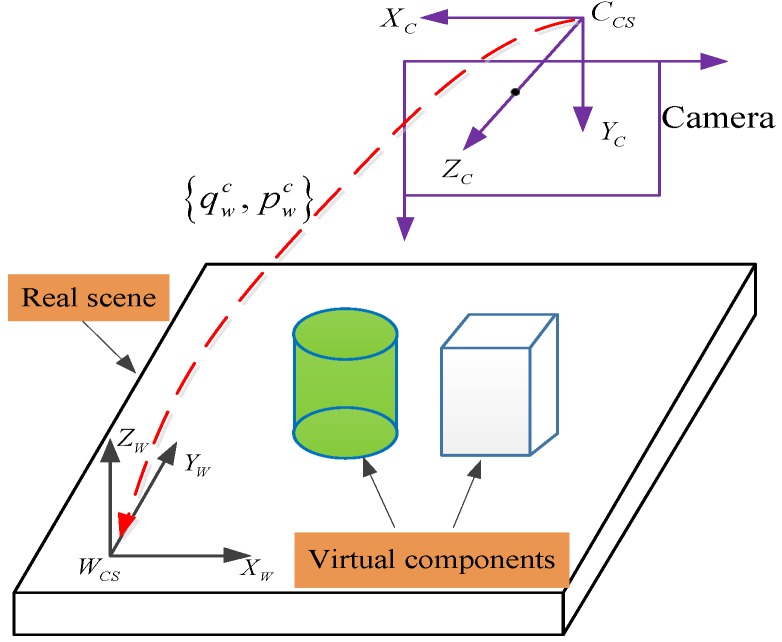
The schematic diagram for AR registration.

Given the proposed real-time 6-DoF pose estimation in mobile devices, the subsequent transformations between the camera frame and the world frame $\left\{q_{w}^{c},\;p_{w}^{c}\right\}$ are obtained. Thus, the virtual components can be rendered to the real scene. Figure 10 illustrates the render schematic in detail, with this transformation $\left\{q_{w}^{c},\;p_{w}^{c}\right\}$, the real scene can be augmented by the virtual components.

**Figure 11:**
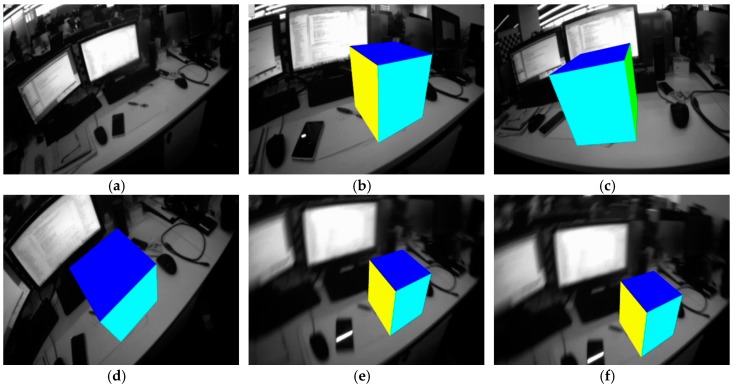
Real-time 6-DoF tracking for mobile AR: (**a**) the original markerless environment; (**b**–**f**) the proposed motion tracking for mobile AR within different viewpoints and conditions.

To verify the proposed real-time motion tracking method for mobile AR, experiments are carried out on a desktop. The original real natural scene is shown in Figure 11a (a screen snapshot from the mobile device). Then, with the proposed real-time 6-DoF tracking method, a virtual cube is augmented to the real scene. Different viewpoints within a loop circle are selected, and the mobile AR performances at different viewpoints are shown from the Figure 11b–d. It is obvious seen that, the virtual object is augmented with fixed locations and orientations. Besides, the performance when the mobile device suffered from strong shakes or motion blur is also evaluated, as shown in Figure 11e,f. Blurred images are captured during intentional shaking of the mobile device, if based on the visual-based motion tracking only, the mobile AR is inclined to collapse due to the unbelievable blurry image. Nevertheless, with the proposed sensor-fusion based tracking approach, the tracking lost phenomenon due to the fast motion blur can be alleviated.

### 4.3. Real-Time Motion Tracking for Mobile VR

VR allows different ways to interact between the user and virtual world. Thus, the real-time tracking of the user’s postures and actions play an important role for a VR system. Moreover, the frame rate for motion tracking in VR puts forward a higher requirement than AR. Otherwise, the latency phenomena would make the users sick. With the proposed adaptive visual-inertial fusion method, a smooth 6-DoF motion tracking for mobile VR can be achieved in real-time.

**Figure 12:**
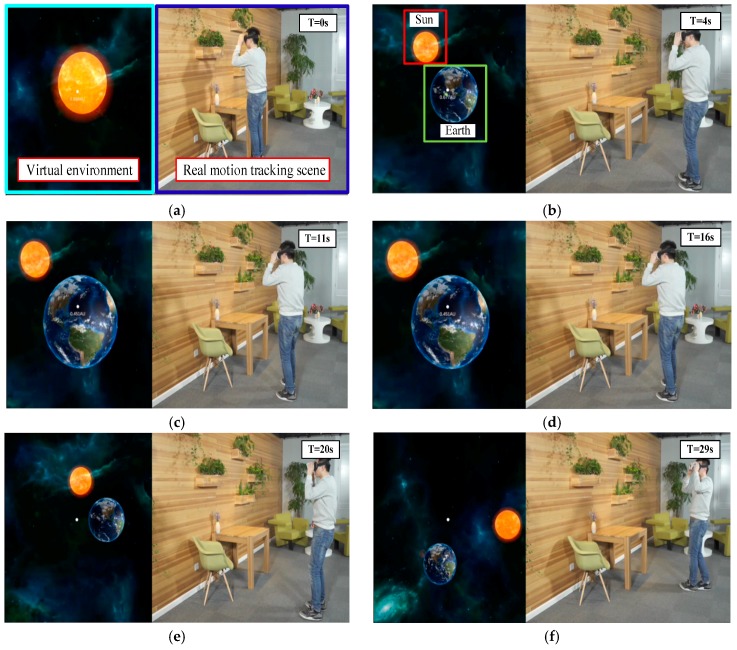
Real-time motion tracking for mobile VR: (**a**,**b**) the virtual and real scene; (**c**,**d**) keep static for jitter testing; (**e**,**f**) a real-time 6-DoF tracking to interact with the virtual scene.

As shown in Figure 12a, the right side is the real-time 6-DoF motion tracking in real scene, and the left side is a corresponding VR environment from the user’s perspective. With the proposed multi-sensor system mounted on the user’s head, the 6-DoF motion of the user in real-time can be perceived. Thus, when the user moves freely in the real scene, the perspective of the virtual scene can change corresponding to the real 6-DoF motion tracking. Thus, with the free motion of the operator, the earth appears in the solar system (as shown in Figure 12b). Given the adaptive visual-inertial fusion, the frame-rate of the self-contained motion tracking can reach real-time performance in a virtual environment. When we keep static at some certain position in real scene about 5 s (T = 11 s–16 s), the virtual scene followed by a stationary state, as shown in Figure 12c,d, with the proposed adaptive filter, the jitter phenomenon can be eliminated in the virtual scene. And then, the location and orientation between the Sun and Earth within the solar system can be adjusted by the free walk in a real scene, as shown in Figure 12e,f. The experimental results also show the feasibility of the proposed tracking method for mobile VR.

## 5. Discussion

Real-time motion tracking is a crucial issue for any AR/VR systems, and there are different methods to realize the tracking performance. In marker-based motion tracking, the system needs to detect and identify the marker, and then calculate the relative pose of the observer. However, the marker need to be stuck on or near the object of interest in advance, and sometimes it is not possible to attach the marker to some certain circumstances. In addition, the marker should remain visible during the mobile AR/VR process, and the tracking is inclined to become corrupt due to the marker being out of view. Similarly, the model-based method is another typical motion tracking method for mobile AR/VR. This tracking method uses a prior model of the environment to be tracked. Usually, this prior knowledge consists of 3D models or 2D templates of the real scene. Nevertheless, the extraction of a robust tracked prior model is not always available, especially in some unorganized natural scenes. With the cost of computer vision decreasing rapidly, the visual-based markerless approach turns out to be a more attractive alternative to perform motion tracking. This method depends on natural features instead of artificial markers or prior models, resulting in a more flexible and effective tracking performance in unprepared environments. However, this markerless tracking method is inclined to collapse when encountering motion blur and fast motion. Moreover, the real-time performance for mobile AR/VR is beyond the traditional video frame-rate, making the visual-based markerless tracking insufficient.

Therefore, the proposed visual-inertial tracking method can work well for mobile AR/VR in an unprepared environment, due to a stronger adaptability than either marker-based or model-based methods. Moreover, with high-frequency measurements from an IMU, the frequency of the motion tracking can be improved compared to the traditional visual-based markerless tracking. Besides, with the help of the temporary IMU integration, the tracking loss phenomenon because of blurred images for mobile AR/VR can be alleviated.

In addition, due to different frequencies of the monocular and IMU measurements, several predicted poses from IMU exist between every adjacent image during the visual-inertial fusion. If the 6-DoF pose from visual-inertial fusion feeds the mobile AR/VR directly, real-time performance can be achieved at the cost of the jitter derived from the IMU prediction. The jitter phenomenon would damage the user experience in mobile AR/VR, thus an adaptive filter framework is proposed in the paper to balance the jitter and latency. It can adjust the filter parameters according to different motion situations, and balance the jitter and latency automatically for mobile AR/VR. For example, if the mobile system is kept stationary, the jitter phenomenon is more obvious for the user than the latency, thus the filter framework can be adjusted to a jitter-filtering scheme by alleviating the jitter phenomenon for mobile AR/VR. Otherwise, another filter stage would be selected when different motion situations are encountered. Thus, a real-time motion tracking by balancing the jitter and latency for mobile AR/VR is obtained from the proposed adaptive filter framework.

Currently, the prototype system of the proposed method is based on an existing smartphone. Thus, the system is more suitable to be installed in a VR/AR headset. Along with the improvement of industrial design and mobile computing capacity in future, this system could be conveniently worn on the user’s body for stronger adaptability.

What is more, in order to improve the tracking performance for mobile AR/VR, an external sensor module containing a wide-angle monocular camera and an inertial sensor is applied in this work. Given similar sensors embedded in current mobile devices, the real-time tracking performance can also carried out within the mobile terminal alone, but it is not robust enough to the external sensor module due to the limiting FOV of the perspective camera.

It is worth noting that the proposed adaptive filter framework is a universal approach for visual-inertial fusion, or some other heterogeneous sensor fusion. Given different configurations of the visual-inertial system, the detailed values of the adaptive parameters should be adjusted slightly, which can be derived from quantitative contrast tests. Moreover, this process is also considered as a parameter calibration for a specific multi-sensor system, and it can provide long-term usage once the corresponding adaptive filter framework is established.

## 6. Conclusions and Future Works

This paper proposes a sensor-fusion based real-time motion tracking approach for mobile AR/VR, which is more powerful than the traditional visual-based markerless tracking ones. Given the real-time and robust posture arriving for mobile AR/VR, a monocular visual-inertial fusion is established in the paper, which can effectively improve the tracking robustness and enhance frame-rate with the help of an inertial sensor. In addition, in order to alleviate the jitter phenomenon within the heterogeneous sensor fusion, an adaptive filter framework is proposed which can adjust the filter weight according to different motion situations, achieving a real-time and smooth motion tracking both for mobile AR and mobile VR. Finally, experiments are carried out in different AR/VR circumstances, the results indicate the robustness and validity of our proposed method.

In this paper, a segmented adaptive framework is defined for a simplifying calculation, and a suboptimal performance is obtained for real-time motion tracking for mobile AR/VR. However, in the tracking performance unstable transitions may exist at the segmented point, thus future work will be done dealing with a more continuous filtering framework for visual-inertial fusion.

## Figures and Tables

**Table 1:** Accuracy of the proposed motion tracking (total length = 1728 cm).

	**Translational Error (cm)**			**Rotational Error (deg)**		
	$T_{x}$	$T_{y}$	$T_{z}$	$R_{x}$	$R_{y}$	$R_{z}$
**Mean error**	5.78	5.67	0.81	0.72	0.67	0.79
**Standard Deviation**	2.98	2.83	1.29	0.37	0.28	0.42
**Maximum error**	10.46	9.83	3.25	1.59	1.41	1.71
